# Myocardial contractility in the stress echo lab: from pathophysiological toy to clinical tool

**DOI:** 10.1186/1476-7120-11-41

**Published:** 2013-11-18

**Authors:** Tonino Bombardini, Monica Zoppè, Quirino Ciampi, Lauro Cortigiani, Eustachio Agricola, Stefano Salvadori, Tiziana Loni, Lorenza Pratali, Eugenio Picano

**Affiliations:** 1 CNR, Institute of Clinical Physiology, Area della Ricerca, Pisa 56124, Italy; 2 Division of Cardiology, Fatebenefratelli Hospital, V.le Principe di Napoli 14, Benevento 82100, Italy; 3 Division of Cardiology, Campo di Marte Hospital, Via dell’Ospedale, 238, Lucca 55100, Italy; 4 Division of Non-Invasive Cardiology, San Raffaele University Hospital, Via Olgettina 58, Milan 20100, Italy

**Keywords:** Force-frequency relation, Pressure-volume relation, Stress echo, Contractility, Heart failure, Wearable contractility sensor, Molecular animation, Bioblender

## Abstract

Up-regulation of Ca^2+^ entry through Ca^2+^ channels by high rates of beating is involved in the frequency-dependent regulation of contractility: this process is crucial in adaptation to exercise and stress and is universally known as force-frequency relation (FFR). Disturbances in calcium handling play a central role in the disturbed contractile function in myocardial failure. Measurements of twitch tension in isolated left-ventricular strips from explanted cardiomyopathic hearts compared with non-failing hearts show flat or biphasic FFR, while it is up-sloping in normal hearts. Starting in 2003 we introduced the FFR measurement in the stress echo lab using the end-systolic pressure (ESP)/End-systolic volume index (ESVi) ratio (the Suga index) at increasing heart rates. We studied a total of 2,031 patients reported in peer-reviewed journals: 483 during exercise, 34 with pacing, 850 with dobutamine and 664 during dipyridamole stress echo. We demonstrated the feasibility of FFR in the stress echo lab, the clinical usefulness of FFR for diagnosing latent contractile dysfunction in apparently normal hearts, and residual contractile reserve in dilated idiopathic and ischemic cardiomyopathy. In 400 patients with left ventricular dysfunction (ejection fraction 30 ± 9%) with negative stress echocardiography results, event-free survival was higher (p < 0.001) in patients with ΔESPVR (the difference between peak and rest end-systolic pressure-volume ratio, ESPVR) ≥ 0.4 mmHg/mL/m^2^. The prognostic stratification of patients was better with FFR, beyond the standard LV ejection fraction evaluation, also in the particular settings of severe mitral regurgitation or diabetics without stress-induced ischemia. In the particular setting of selection of heart transplant donors, the stress echo FFR was able to correctly select 34 marginal donor hearts efficiently transplanted in emergency recipients. Starting in 2007, we introduced an operator-independent cutaneous sensor to monitor the FFR: the force is quantified as the sensed pre-ejection myocardial vibration amplitude. We demonstrated that the sensor-derived force changes at increasing heart rates are highly related with both max dP/dt in animal models, and with the stress echo FFR in 220 humans, opening a new window for pervasive cardiac heart failure monitoring in telemedicine systems.

## Introduction

The assessment of left ventricular contractility (usually obtained in the daily routine through gross proxies such as ejection fraction and regional wall motion) is an old dream for the cardiologist, but until recently it has also been a methodological nightmare. The pressure-volume relationship is a conceptually immaculate way to assess contractility and is extensively used in the experimental setting for animal studies, but only rarely in the clinical arena, and then only for research purposes in complex, technically demanding studies requiring invasive cardiac catheterization and maneuvers in the cath lab, with the attending risks of invasivity, contrast injection and radiation exposure. In the last 10 years, left ventricular contractility was brought into the noninvasive stress imaging lab, and particularly in the stress echo lab, where with minimal extra-computing time and virtually no extra-imaging time, left ventricular contractility can be measured at baseline and during stress with simple raw measurements of end-systolic pressure (by tonometry or cuff sphygmomanometer) and end-systolic volume (by 2D or, even better, real time 3-D echo). In this way, left ventricular contractility no longer “cuts the airy way” of pathophysiology but has entered the real world of clinical cardiology. This review will discuss the pathophysiological basis, experimental evidence, and clinical data supporting a more extensive use of contractile reserve in key diagnostic domains such as identification of coronary artery stenosis, myocardial viability and initial occult cardiomyopathy.

### The pathophysiological basis

**Figure 1:**
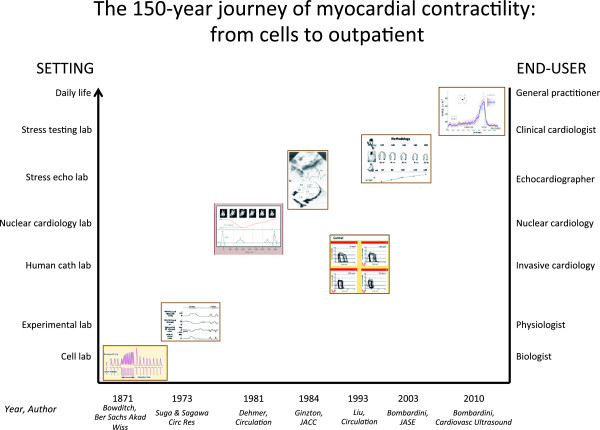
The 150-year-long journey of myocardial contractility from bench to bedside, from catheterization lab to cardiac imaging techniques, and from imaging-dependent to imaging-independent assessment.

**Figure 2:**
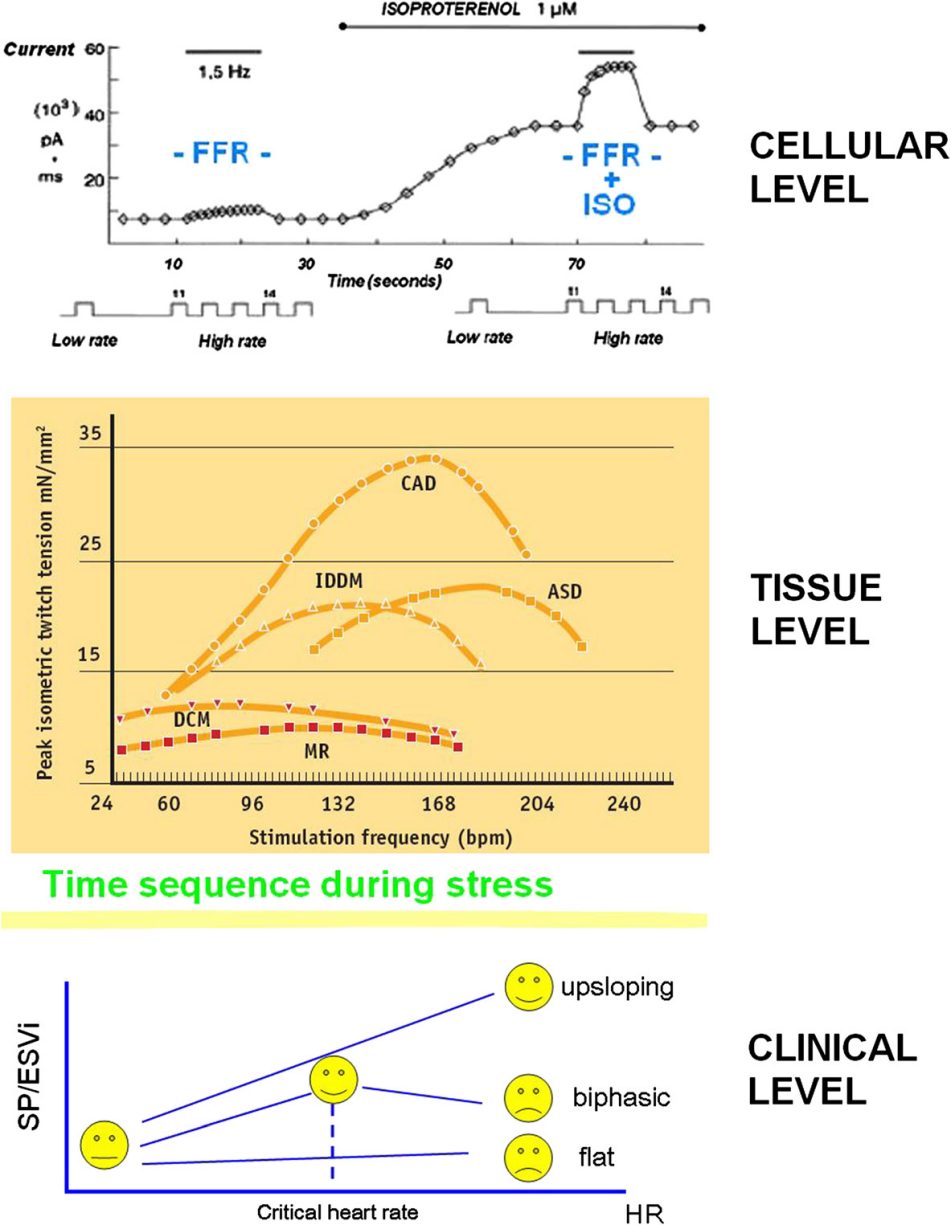
**FFR, from myocardial strips to the echo lab.** Isolated cardiomyocytes (upper panel): upregulation of Ca^2+^ entry through Ca^2+^ channels by high rates of beating is involved in the frequency-dependent regulation of contractility. The effect of increasing contractility by increasing heart rate ("pure" Bowditch treppe) is intrinsic to myocardium and takes a few seconds to occur, while the β-adrenergic amplification of the force-frequency relation (FFR) takes longer, i.e., 30–40 seconds, the time it takes for β-receptor activation and cAMP synthesis (on the right: FFR + ISO). (Modified from: Piot C, Circ 1996 [3]). Middle panel: measurements of twitch tension in isolated left-ventricular strips from explanted cardiomyopathic hearts: the FFR of these failing groups both exhibit a *negative treppe* at contraction frequencies above about 100 bpm. The contraction frequency at which the FFR begins its descending limb ("optimum stimulation frequency") declines progressively in the order: ASD (atrial septal defect), CAD (coronary artery disease), IDDM (diabetic myopathy), MR (mitral regurgitation), DCM (dilated cardiomyopathy). (Modified from: Mulieri LA. R.A. Howarth Ed. 1997). Lower panel: time sequence during stress echo; the force-frequency relation is built off line. The force-frequency relation is defined as up-sloping when the peak stress systolic pressure/end-systolic volume index (SP/ESV index) is higher than baseline and intermediate stress values; biphasic, with an initial up-sloping followed by a later down-sloping trend, when the peak stress SP/ESV index is lower than intermediate stress values; flat or negative, when the peak stress SP/ESV index is equal to or lower than baseline stress values. The critical heart rate (or optimum stimulation frequency) is the human counterpart of the treppe phenomenon in isolated myocardial strips (Modified from Bombardini T, CU 2005 [1]).

Contractility is the intrinsic ability of heart muscle to generate force and to shorten, independently of changes in the preload or afterload with fixed heart rates. At the molecular level the crux of the contractile process lies in the changing concentrations of Ca^2+^ ions in the myocardial cytosol. Ca^2+^ ions enter through the calcium channel that opens in response to the wave of depolarization that travels along the sarcolemma. These Ca^2+^ ions “trigger” the release of more calcium from the sarcoplasmic reticulum (SR) and thereby initiate a contraction-relaxation cycle [1]. An increased stimulation rate increases the force of contraction: the explanation is repetitive Ca^2+^ entry with each depolarization, and hence, an accumulation of cytosolic calcium. As the heart fails, there is a change in the gene expression from the normal adult pattern to that of fetal life with an inversion of the normal positive slope of the force-frequency relation [2]: systolic calcium release and diastolic calcium reuptake process is lowered at the basal state, and instead of accelerating for increasing heart rates, slows down [3] (Additional files 1, 2, 3, 4, 5). In the past, several attempts were made to transfer the pure physiological concept of contractility, expressed in the isolated myocardial fiber by the maximal velocity of contraction of unloaded muscle fiber (Vmax), to the in vivo beating heart. Suga and Sagawa achieved this aim by measuring pressure/volume loops in the intact heart: during a positive inotropic intervention, the pressure volume loop reflects a smaller end-systolic volume and a higher end-systolic pressure [4] (Figure 1). The pressure-volume relationship is the most reliable index for assessing myocardial contractility in the intact circulation and is almost insensitive to changes in preload and afterload. Non-invasive measurement of the end-systolic pressure-volume-ratio (ESPVR) for increasing heart rates during stress in the echo is the practical answer to this new clinical demand due to a current dramatic increase in the number of heart failure patients [5]. Ten years ago ΔESPVR (or simpler PVR, pressure-volume relation) was introduced in the stress echo lab as a measure of the heart rate-dependent changes in contractility [6], associated or not with adrenergic stimulation [7,8] (Figure 2).

### The stress echo methodology

**Figure 3:**
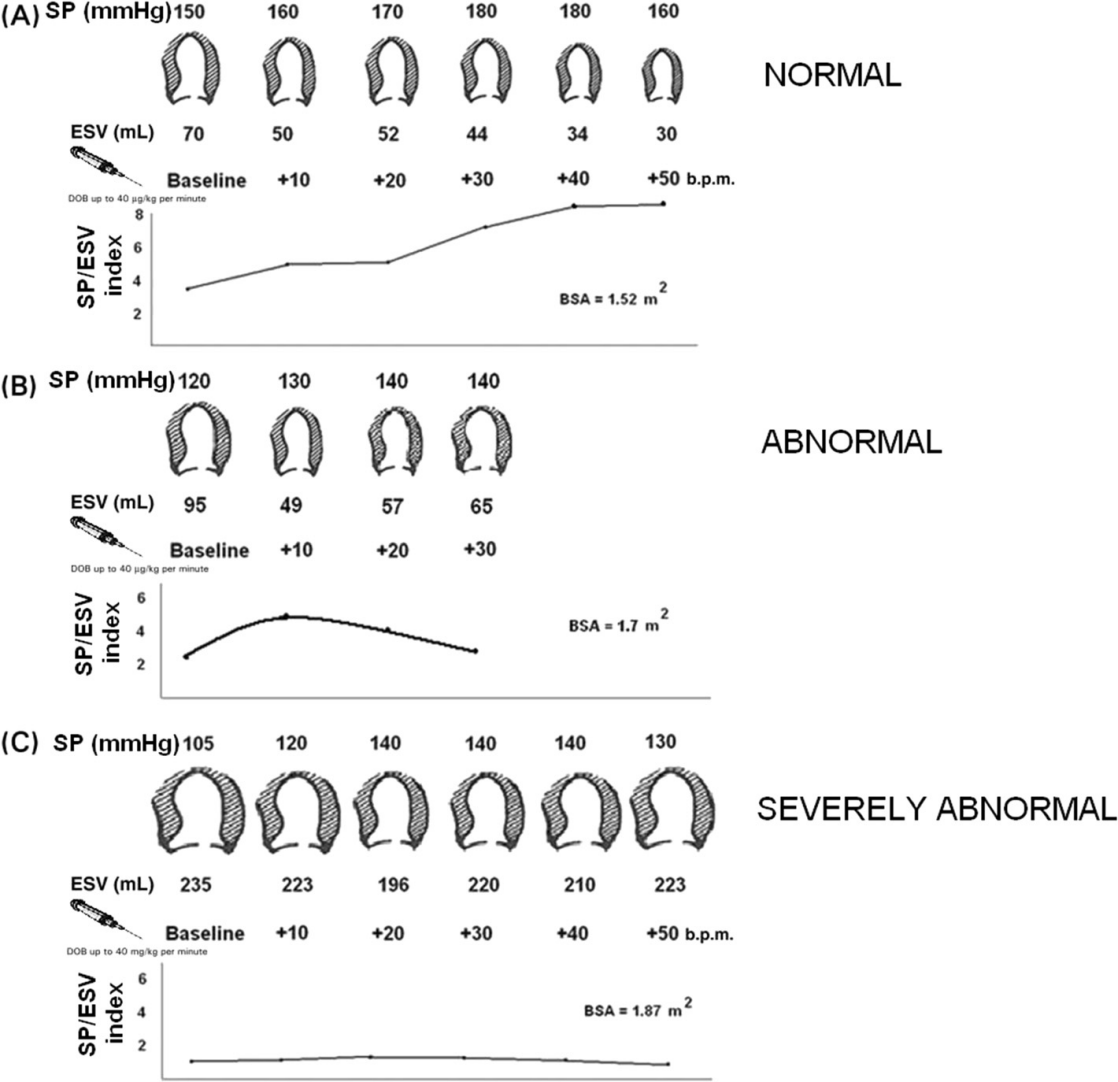
**Methodology of the force-frequency relation with stress echo.** Left, from upper to lower rows: systolic blood pressure (SP) by cuff sphygmomanometer (first row); LV end-systolic volumes (ESV) calculated with the biplane Simpson method (second row); heart rate increase (b.p.m.) during dobutamine infusion (third row); in the lowest row, the force-frequency relation built off-line with the values recorded at baseline (second column), and at different steps (third, fourth, fifth columns) up to peak exercise (seventh column). **(A)** Normal subject. An increased heart rate is accompanied by an increased systolic pressure with smaller end-systolic volumes (normal up-sloping PVR). **(B)** A subject with LV dysfunction (EF% = 32%) without dilation, no stress-induced ischemia. The PVR was biphasic, with an initial up-sloping followed by a later down-sloping trend, the critical heart rate (90 b.p.m.) was the heart rate beyond which SP/ESV index declined by 5%. The test was stopped at 20 gammas due to limiting symptoms (dyspnea). **(C)** A subject with post-MI depressed baseline LV function (EF% = 30%). An increased heart rate at peak exercise is accompanied by no changes in end-systolic volumes (abnormal flat PVR). (Modified from Grosu A, EHJ 2005). BSA = Body surface area; ESV = End-systolic volume; PVR = Pressure-volume relation; SP = Systolic pressure; SP/ESV = Systolic pressure/end-systolic volume ratio.

The evaluation of LV contractility is potentially amenable to non-invasive assessment in the cardiac imaging lab, since cuff systolic pressure is a proxy of end-systolic pressure (in absence of aortic stenosis) and end-systolic volumes can be evaluated by radionuclide ventriculography [9] or – with better spatial and temporal resolutions – by 2D-echo, both at baseline and during stress. To build the force-frequency relation, the force is determined at each step as the ratio of the systolic pressure (cuff sphygmomanometer)/end-systolic volume index (biplane Simpson rule/body surface area) (Additional files 6 and 7). The LV end-systolic volume is obtained from apical four-chamber and two-chamber view using the biplane Simpson method [6-8]. The LV end-systolic volume is assessed at rest and at peak stress and normalized by dividing it by body surface area. Only representative cycles with optimal endocardial visualization are measured and the average of three measurements are taken. The endocardial border is traced, excluding the papillary muscles. The frame with the smallest left ventricular cavity is considered to be the end-systolic frame. The Left Ventricular End-Systolic Pressure (LVESP, mmHg) is obtained as LVESP = 0.9 * Systolic Blood Pressure (mmHg) [10]. The end-systolic pressure-volume-ratio (ESPVR mmHg/mL/m^2^) is obtained as the ratio of the end-systolic pressure [ESP] to the LV end-systolic volume indexed for body surface area. The ESPVR is determined at rest and at peak stress. The ΔESPVR (or simpler PVR, pressure-volume relation) is calculated as the variation between rest and peak stress ESPVR. The rest ESPVR, the peak stress ESPVR and the ΔESPVR are built offline [6-8,11]. The force-frequency relation is defined as up-sloping when peak stress ESPVR is higher than baseline and intermediate stress values (Figure 3 upper panel); biphasic, with an initial up-sloping followed by a later down-sloping trend, when peak stress ESPVR is lower than intermediate stress values [12,13] (Figure 3 middle panel), flat or negative, when peak stress ESPVR is equal to or lower than baseline stress values (Figure 3 lower panel). The conventionally used stimuli in stress echocardiography act via different mechanisms to detect myocardial contractility [14]: exercise acts via heart rate increase and endogenous catecholamine stimulation during exercise [6] (Additional files 6 and 7); dobutamine acts via heart rate increase and exogenous adrenergic stimulation [7]; pacemaker stress is applied in patients with permanent PM: external programming of the permanent PM induces a controlled change in heart rate, with no adrenergic stimulation [8]. Dipyridamole stress causes a three- to fourfold increase in coronary blood flow and increases contractile function by the Gregg effect [11].

### Feasibility and clinical applications

**Figure 4:**
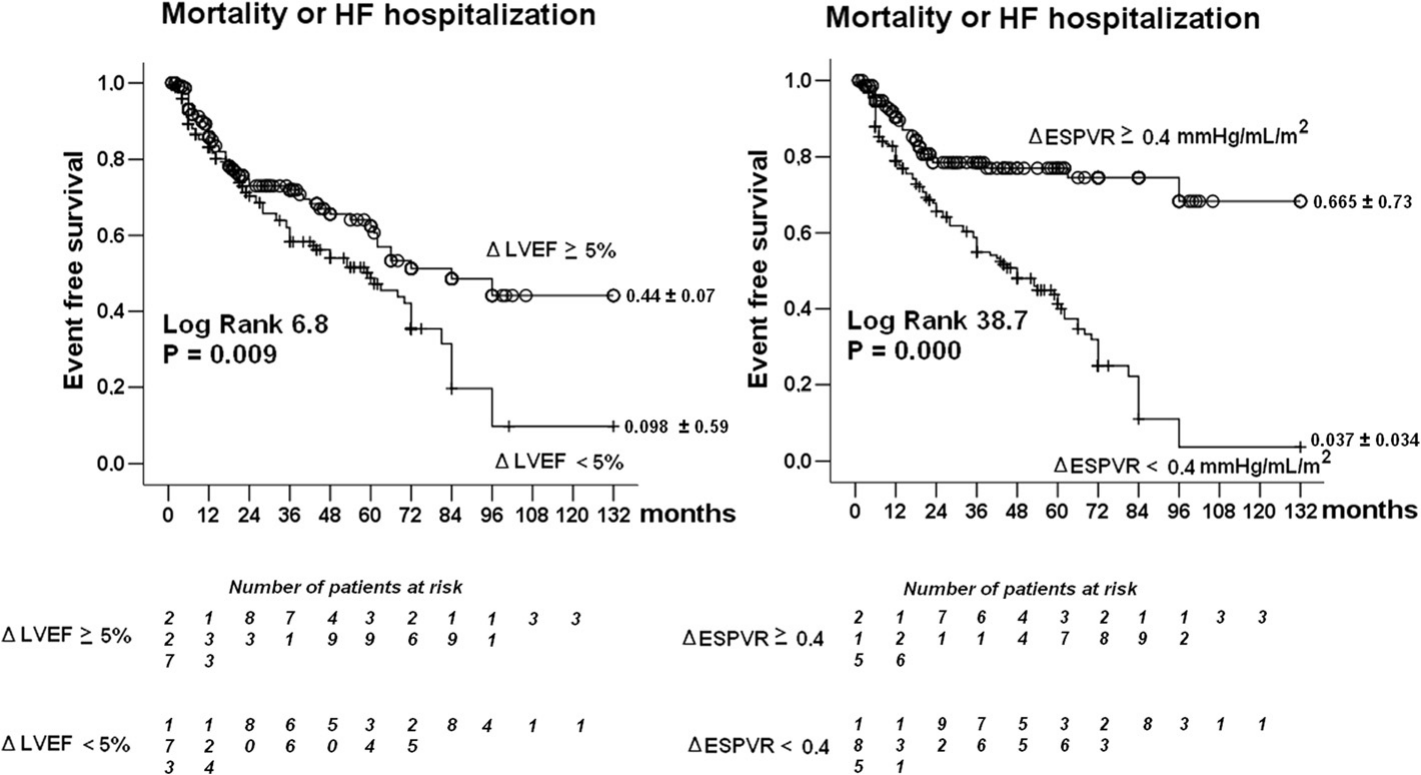
**Prognostic predictors – *Left panel.*** Kaplan-Meier survival curves (considering combined death or HF hospitalization as an endpoint) in medically treated patients stratified according to the presence of stress ΔLVEF ≥ 5% vs rest as cut-off value. *Right panel.* Kaplan-Meier survival curves (considering combined death or HF hospitalization as an endpoint) in medically treated patients stratified according to the presence of stress ΔESPVR ≥ 0.4 mmHg/mL/m2 vs rest as cut-off value.

**Figure 5:**
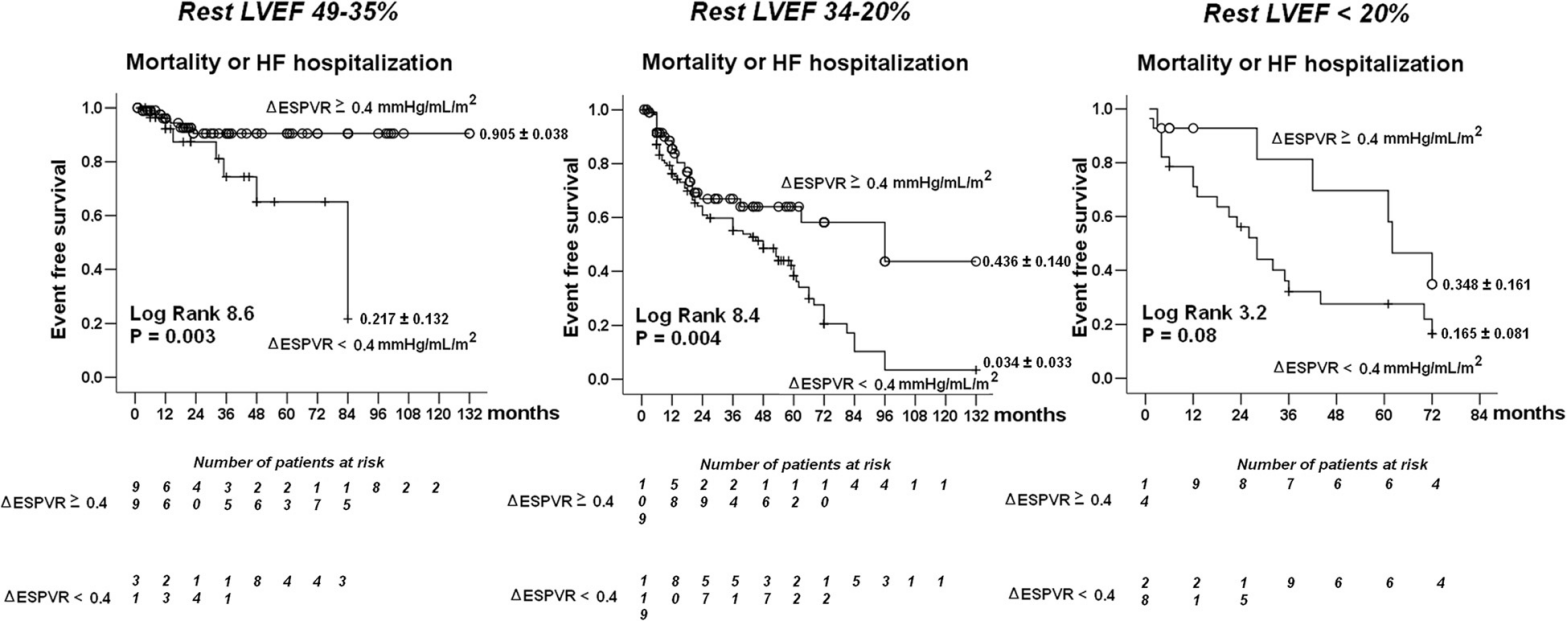
**Survival in patients with negative stress echo and different rest LV dysfunction.** Kaplan-Meier survival curves (considering combined death or HF hospitalization as an endpoint) in medically treated patients stratified according to the presence of stress end-systolic pressure-volume relation-based contractile reserve (**Δ**ESPVR ≥ 0.4 mmHg/mL/m2 vs rest as cut-off value) in patient with moderate (left panel), severe (middle panel), or extreme (right panel) rest LV dysfunction.

**Figure 6:**
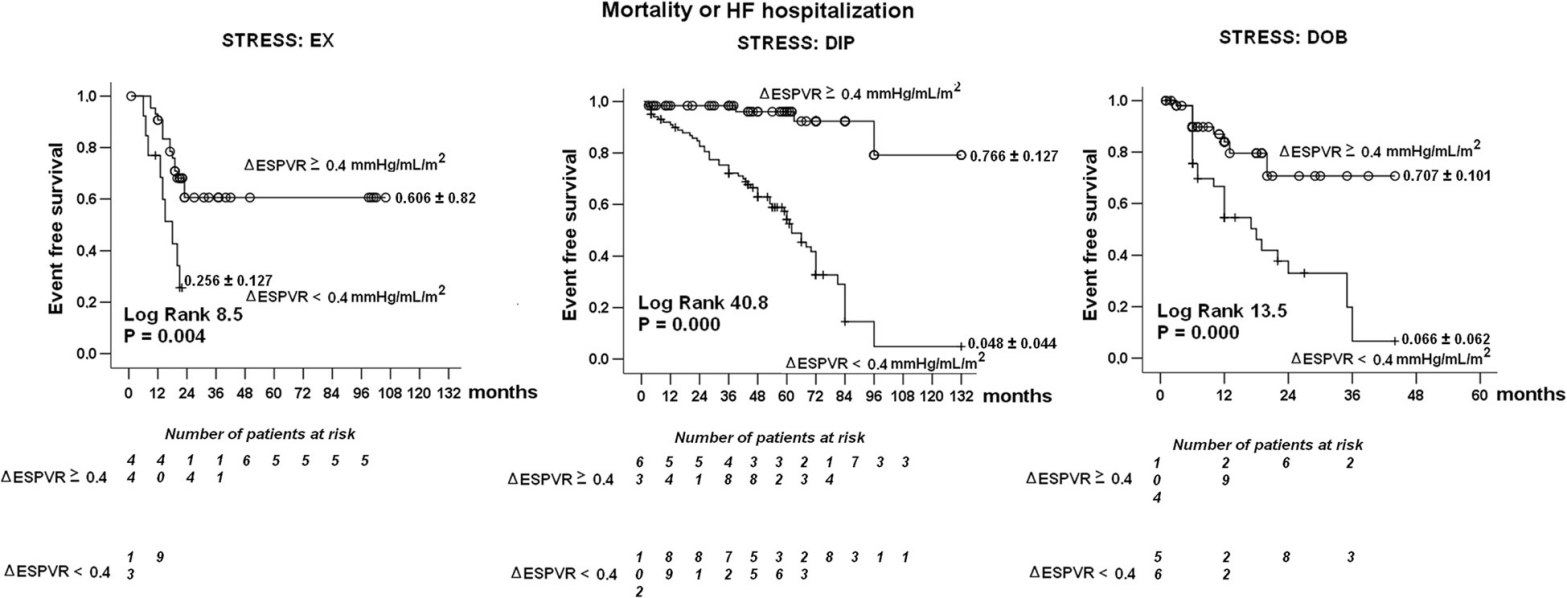
**Survival in patients with negative stress echo according to the employed stress.** Kaplan-Meier survival curves (considering combined death or HF hospitalization as an endpoint) in medically treated patients stratified according to the presence of stress end-systolic pressure-volume relation-based contractile reserve (**Δ**ESPVR ≥ 0.4 mmHg/mL/m^2^ vs rest as cutoff value). *Left panel*, patients (n = 57) stratified according to exercise stress echo. *Middle panel*, patients (n = 165) stratified according to dipyridamole stress echo. *Right panel*, patients (n = 160) stratified according to dobutamine stress echo.

Starting in 2003 we introduced the FFR measurement in the stress echo lab using the ESPVR (the Suga index) at increasing heart rates. We studied a total of 2,031 reported patients in peer-reviewed journals: 483 with exercise stress, 34 with pacing stress, 850 with dobutamine stress echo, 664 with dipyridamole stress echo [6-8,11,15-27]. The feasibility and the reproducibility of measurements was consistently reported as very high in all studies, with all forms of stresses, and in different patient populations – from ischemic or idiopathic dilated cardiomyopathy to severe mitral insufficiency (Table 1). We did not study patients with aortic stenosis. Exercise stress echocardiography is strictly contraindicated in symptomatic patients. In asymptomatic patients with aortic stenosis the ΔESPVR could be calculated during stress, but the trans aortic systolic pressure drop should be added to the brachial systolic pressure to correctly calculate the ESPVR both at rest and at peak stress. Different normal vs abnormal ΔESPVR cut-off-values have been observed for different stressors such as exercise [6,15,18,23,25,27,28], subclinical heart disease in diabetes [19,28], to donor hearts not eligible for heart transplant [17,26,29]. A systematic review of the literature with meta-analysis was needed to identify all published papers examining the use of ΔESPVR (PVR) for the prediction of cardiac death and/or heart failure hospitalization in patients with resting LV dysfunction. In eight studies summarized in Table 2, the prognostic information was available, enrolling patients with left ventricular dysfunction and non-inducible ischemia at stress echocardiography with different selection criteria – from suspected coronary artery disease to dilated cardiomyopathy. The optimal cut-off for prognostically relevant events was different with the different stresses, the different populations, and the different prognostic end-points taken into consideration (Table 3). In particular, we identified six [7,8,15,23-25] eligible studies which provide information on the incidence of cardiac death and/or heart failure hospitalization in heart failure patients. During a median follow-up of 19 months (interquartile range 6–48), 53 deaths (49 cardiac, 4 non-cardiac), and 83 HF-related hospitalizations occurred. Of the 83 patients with hospitalizations for heart failure, 5 underwent heart transplant, and 20 a CRT-ICD implant. Eighteen patients underwent revascularization at follow-up (5 with viability response). With a receiver-operating characteristic analysis, ΔESPVR < 0.4 mmHg/ml/m^2^ (area under the curve 0.758, 95% CI 0.708 to 0.803; positive predictive value 55%, negative predictive value 84%), was the best predictor of future primary cardiovascular events (composite of overall death and heart failure hospitalization) (Figure 4). Resting LVEF, a powerful predictor of future cardiovascular events, showed a significant cutoff value of < 30% (area under the curve 0.730, 95% CI 0.680-0.780; positive predictive value 50%, negative predictive value 81%). A viability response (WMSI decrease during stress) showed a trend toward prediction of cardiac events with a significant cutoff value of −0.235 (AUC 0.567, 95% CI 0.507 to 0.627; positive predictive value 42%, negative predictive value 72%). The same contractile reserve cutoff (ΔESPVR < 0.4 mmHg/mL/m^2^ ) was also effective in stratifying the risk in the subgroups with progressively more severe baseline dysfunction (Figure 5), and in the subgroups with different stimuli used in stress echocardiography (Figure 6).

**Table 1:** The spectrum of diagnostic applications of contractility in the stress echo lab

	**Author, year**	**Stress**	**Pts (n)**	**Inclusion criteria**	**Feasibility**	**Intra observer variability**	**Inter observer variability**	**Results**
**CAD diagnosis**								
	Bombardini, JASE 2003 [6]	EX	50	Consecutive pts	100% (by selection)	NA	NA	Biphasic PVR (<2 mmHg/ml/m^2^) with contractility loss at ischemia
	Grosu, Eur Heart J 2005 [7]	DOB	100	Abnormal LV function	100% (by selection)	Within 2 SD*	Within 2 SD*	Biphasic PVR (<2 mmHg/ml/m^2^) with contractility loss at ischemia
	Bombardini, Eur J Heart Fail 2005 [8]	Pacing	26	Permanent PM	100% (by selection)	NA	NA	Biphasic PVR (<2 mmHg/ml/m^2^) with contractility loss at ischemia
	Bombardini, Int J Cardiol 2013 [8]	DIP	111	Normal LV function	100% (by selection)	NA	NA	Negative (<0 mmHg/ml/m^2^) PVR in positive tests
**Contractile reserve in DCM**								
	Otasevic, Eur J Heart Fail 2005 [21]	DOB	24	Scheduled endomyocardial biopsy	88%	NA	NA	Flat PVR (< 0.4 mmHg/ml/m^2^) in increased myocyte diameter
	Cortigiani, Heart 2009 [19]	DOB	233	Diabetics with negative stress	100% (by selection)	8%	11%	Peak ESPVR < 28 mmHg/ml/m^2^ as events predictor
	Ciampi, JASE 2010 [22]	DOB	37	CHF center	89%	Within 2 SD*	Within 2 SD*	Flat PVR (< 0.5 mmHg/ml/m^2^) in NYHA > II with increased BNP
	Bombardini, Biomed Res Int 2013 [25]	EX	18	Polycentric	96%	Within 2	Within 2	Flat-negative
		DIP	146	study		SD*	SD*	PVR (< 0.5 mmHg/ml/m^2^) in NYHA > II
		DOB	58					
**Contractile reserve in ischemic DC**								
	Bombardini, Biomed Res Int 2013 [25]	EX	36	Polycentric study	96%	Within 2 SD*	Within 2 SD*	Positive correlation (R = 0.56, p = 0.000) with oxygen consumption
	Bombardini, CU 2007 [27]	EX	52	Comparisons with wearable contractility sensor		NA	NA	Post exercise contractility overshoot in abnormal flat PVR
**Contractile reserve in MR**								
	Agricola, Am J Cardiol 2005 [18]	EX	63	Normal LV function in severe MR	100%	5%	4%	Blunted PVR (< 2.1 mmHg/ml/m^2^) in stress induced pulmonary hypertension
**Contractile reserve in diabetes**								
	Jellis, Circ CI 2010 [28]	EX	167	Apparently healthy type 2 diabetics	100% by selection	NA	NA	PVR ≤ 12 mmHg/ml/m^2^ in subclinical myocardial disease
**Contractile reserve in potential heart donors**								
	Leone, IJHLT 2009 [26]	DIP	18	Older donor hearts	100%	NA	NA	CAD / myocardial disease of non transplanted hearts with abnormal negative (< 0 mmHg/ml/m^2^) PVR
	Bombardini, JASE 2011 [17]	DIP	39	Older donor hearts	100%	NA	NA	Normal coronary arteries and post-TX LV function in 19 hearts with up-sloping (> 0 mmHg/ml/m^2^) PVR
	Bombardini, CU 2103 [29]	DIP	6	Stunned donor hearts	100%	NA	NA	TX of hearts with viability response and positive (> 0 mmHg/ml/m^2^) PVR

CAD = Coronary artery disease; CHF = Chronic heart failure; DC = Dilated ischemic cardiomyopathy; DCM = Idiopathic dilated cardiomyopathy; DIP = Dipyridamole; DOB = Dobutamine; EX = Exercise; LV = Left ventricle; MR = Mitral regurgitation; NA = Not available; PM = Pace-maker; PVR = Pressure-volume relation; TX = Heart transplant. *Both for intraobserver and interobserver analysis, > 95% of the differences were between *d −*2 SD and *d* +2 SD, as described by Bland and Altman.

**Table 2:** The prognostic value of contractility in the stress echo lab

**Author, year**	**Stress**	**Pts (n)**	**Inclusion criteria**	**LVEF**	**Median follow-up (months)**	**Best prognostic PVR cut-off**	**Considered events**	**Events n (%)**	**PPV**	**NPV**
Grosu A et al., 2005 [7]	DOB	95	Medically treated pts with LV dysfunction	40±15%	18	<0.2 mmHg/ml/m^2^	Death, HF	18 (19%)	77%	89%
Otasevic P et al., 2006 [20]	DOB	59	Idiopathic dilated cardiomyopathy	19±8%	60	<0.33 mmHg/ml/m^2^	Death	27 (46%)	62%	84%
Bombardini T et al., 2008 [15]	EX	99	Medically treated pts with negative stress	47±14%	21	<2.2 mmHg/ml/m^2^	Death, HF, ↑NYHA	29 (29%)	62%	98%
Agricola E et al., 2008 [23]	EX	37	Functional MR in LV dysfunction	29±7%	20	<0.4 mmHg/ml/m^2^	Death	8 (22%)	35%	93%
Ciampi Q et al., 2010 [24]	DOB	72	Identification of CRT responders	26±6%	12	<0.7 mmHg/ml/m^2^	Death, HF	22 (31%)	40%	91%
Bombardini T et al., 2011 [17]	DIP	16	Recipients of older donor hearts	56±6%	14	<0 mmHg/ml/m^2^	Death	2 (13%)	50%	94%
Bombardini T et al., 2013 [25]	EX	172	Followed-up Pts with negative stress	53±14%	32	<1.34 mmHg/ml/m^2^	Death, HF	29	55%	96%
	DIP	482		50±17%	21	<0.46 mmHg/ml/m^2^	Death, HF	68	24%	97%
	DOB	237		39±15%	6	<0.56 mmHg/ml/m^2^	Death, HF	37	40%	94%
Cortigiani L et al., 2009 [19]	DOB	233	Diabetics with negative stress	52±10%	18	<12 mmHg/ml/m^2^	Death, PCI/CABG	62 (27%)	36%	83%

CABG = Coronary artery by-pass grafting; CAD = Coronary artery disease; CRT = Cardiac resynchronization therapy; DIP = Dipyridamole; DOB = Dobutamine; EX = Exercise; HF = Heart failure; LV = Left ventricle; LVEF = Left ventricular ejection fraction; MR = Mitral regurgitation; NPV = Negative predictive value; PCI = Percutaneous coronary intervention; PPV = Positive predictive value; PVR = Pressure-volume relation.

**Table 3:** PVR cut-off for different targets

**Author, year**	**Stress**	**PVR cutoff (mmHg/mL/m**^**2**^**)**	**Clinical target**
Bombardini et al., JASE 2003 [6]	EX	<4.7	Patients vs normal
Agricola et al., Am J Cardiol 2005 [18]	EX	< 2.1	Pulmonary hypertension in MR
Bombardini et al., Int J Cardiol 2008 [15]	EX	< 2.2	Prognosis in negative stress
Agricola et al., Int J Cardiol 2008 [23]	EX	< 0.4	Prognosis in MR in DCM/DC
Jellis et al., Circ Cl 2010 [28]	EX	≤ 12	Subclinical heart disease in diabetic type 2
Grosu et al., Eur Heart J 2005 [7]	DOB	< 0.2	Prognosis in DCM/DC
Otasevic et al., Heart 2006 [20]	DOB	< 0.4	Prognosis in DCM
Ciampi et al., JASE 2010 [22]	DOB	> 1.4	Exercise tolerance in CHF
Ciampi et al., Am Heart J 2010 [24]	DOB	> 0.7	CRT responders
Otasevic et al., Eur J Heart Fail 2005 [21]	DIP	< 0.6	Prognosis in DCM
Bombardini et al., JASE 2011 [17]	DIP	≤ 0	Subclinical heart disease in heart donors
Bombardini et al., Eur J Heart Fail 2005 [8]	PM	< 2.5	Patients vs normal

CHF = Chronic heart failure; CRT = Cardiac resynchronization therapy; DC = Dilated ischemic cardiomyopathy; DCM = Idiopathic dilated cardiomyopathy; DIP = Dipyridamole; DOB = Dobutamine; EX = Exercise; MR = Mitral regurgitation; PM = Pace-maker; PVR = Pressure-volume relation.

### Contractile reserve and coronary flow reserve

In patients with normal baseline left ventricular function and negative stress echo by standard wall motion criteria, the evaluation of contractile reserve provided additional diagnostic information. Lack of contractile reserve can identify minor forms of anatomically significant coronary artery disease (CAD) that are unable to give the absolute subendocardial underperfusion necessary to induce true regional ischemia and wall motion abnormalities. In patients with negative stress and significant (≥ 50%) obstructive CAD, the contractile reserve is attenuated in proportion to the severity of stenosis [11]. A negative contractile reserve, especially when combined with reduced coronary flow reserve (CFR), predicts angiographic evidence of coronary stenosis in patients with normal LV function and negative (not normal!) dipyridamole stress echocardiograms. In patients with DCM and microvascular dysfunction, the CFR was directly related to the contractile reserve; global heart function is increased after a vasodilator stimulus due to the erectile properties of normal myocardium.

### Contractile reserve, strain and strain rate

Galderisi et al. [30] assessed strain rate (SR) imaging during dobutamine stress, to investigate inotropic response in patients with type 2 diabetes mellitus and without coronary artery disease. At every level of dobutamine, strain increased with increasing heart rate (HR) in either group (both P < .0001), but the slope of the overall relation between HR and strain was lower in diabetics (b = −0.08) than in controls (b = −0.14) (P < .01). The blunted slope of the relation between HR and regional strain suggests the impairment of the myocardial force-frequency relation, indicating altered contractile reserve in uncomplicated diabetes.

In an another paper by Jellis et al. [28], in subclinical diabetic heart disease the ΔESPVR is relatively poorly associated with standard echocardiographic measures of myocardial dysfunction such as calibrated integrated backscatter, strain, and strain rate. The disparity between the ΔESPVR and echocardiographic parameters of myocardial dysfunction is likely because individual markers of LV dysfunction identify different pathological processes occurring concurrently within the spectrum of diabetic heart disease. These processes may include interstitial fibrosis, microvascular disease and cardiac autonomic neuropathy; this may enable targeted therapy to be instituted sooner in the disease process.

### Limitations

Limitations of this noninvasive study include that peak cuff systolic pressure was used as a surrogate marker of end-systolic pressure. However, there is a close relationship between these two variables, and any sampling error is duplicated between rest and stress values, thereby not affecting the overall ratio [28]. Calcium channel blockers and β-blockers were not withdrawn before testing in 227 (57%) of patients, and there may still have been blunting of the systolic blood pressure. However, in this “real world” study this was unavoidable as it was thought to be unethical to withhold proven medical therapy from subjects.

### Future developments: imaging-independent, sensor-based contractility

In recent years, the FFR has been transferred from the experimental lab to the clinical arena, and in particular within the clinical arena from the cardiac catheterization to the stress echo lab, with an obvious step-up in interest and applicability. Ideally, the non-invasive, imaging-independent, objective assessment of FFR would greatly further enhance its practical appeal (Figure 1). A new cutaneous force-frequency relation recording system has recently been validated in the stress echo lab, based on heart sound amplitude and timing variations at increasing heart rates [16]. Force-frequency relation recording is feasible with a single precordial vibration sensor connected to a standard ECG monitoring electrode, and quantitatively documents left ventricular contractility changes in a totally automatic, operator-independent fashion. Sensor built force-frequency relation is strictly related to the standard stress echo built FFR. Echocardiography uses artificially generated cardiac reflections. The isovolumic systolic force sensor simply records naturally generated heart vibrations. The FFR is built online. The system is portable, and the remote transmission of the FFR slope and shape is feasible and simple [27]. This approach is extendable to daily physiological exercise and could be potentially attractive in home monitoring systems. To fully establish the advantages and limits of this new method, comparisons with left ventricular pressure-volume loops in humans and multicenter study data are needed.

## Conclusions

Patients with abnormal rest LVEF and negative stress echocardiography by conventional regional wall motion criteria may still experience an adverse outcome in the long-term follow-up, which can be identified by ΔESPVR, providing a prognostic stratification in addition to that given by regional wall motion abnormalities and LVEF. Despite exercise, pharmacological and pacing are different ischemic stressors in terms of cardiac, systemic and coronary hemodynamics, the same contractile reserve cut-off value of ΔESPVR stratifies patients equally well for non-ischemic events at follow-up. In this task, the contractility information complements clinical, coronary flow reserve (CFR), and therapeutic information, allowing stratification of the different “shades of white” of a negative response, with very benign prognosis in subjects with normal resting function, preserved resting LV function, normal (> 2.0) CFR on LAD, and normal test studied off-therapy, with hard events rate < 1% per year. At the other end of the spectrum are subjects with subclinical tests, undergoing therapy with impaired resting LV function, blunted contractile reserve, reduced CFR and hard-event rate > 3% (Table 4). Contractility assessment may be one of the parameters useful for assessing “wolves in sheep’s clothing” -- patients with suboptimal prognosis in spite of negative stress echo by wall motion criteria. On the basis of available, still largely immature but growing evidence, the assessment of contractile reserve with ΔESPVR might provide clinically incremental information in three main subsets: 1) patients with known or suspected coronary artery disease with test negativity by regional wall motion analysis (in whom higher contractile reserve means better prognosis, even in extreme conditions such as selection of potential aged heart donors); 2) patients with ischemic or non-ischemic left ventricular dysfunction without functional recovery with regional or global wall motion analysis, in whom higher contractile reserve means better prognosis; 3) patients with valvular heart disease (for instance, asymptomatic severe mitral insufficiency) or initial cardiomyopathy (for instance in diabetics, hypertensives or oncology patients undergoing potentially cardio-toxic chemotherapy), in whom the detection of initial, occult, yet clinically important forms of left ventricular dysfunction can be important when conventional parameters of left ventricular function (such as regional wall motion or ejection fraction) look normal during stress. The long 150-year journey of myocardial contractility from the physiology lab to bedside is over. The clinical adventure begins now, and cardiologists should make the most of the clinical dividends promised by this fascinating variable.

**Table 4:** Negative stress echo by wall motion criteria: further prognostic refinement

**RISK**	**Not-so-low**	**Very low**
**Dose/load**	**Submaximal**	**Maximal**
Resting ejection fraction	<40%	>50%
Anti-ischemic therapy	+	-
Coronary flow reserve (ratio)	<2.0	>2.0
Contractile reserve (ΔESPVR, mmHg/mL/m^2^)	<0.4	>0.4
Hard events/year	>3%	<1%

ΔESPVR (or simpler PVR, pressure-volume relation): the difference between peak stress and rest ESPVR.

## Abbreviations

BSA: Body surface area; CABG: Coronary artery by-pass grafting; CAD: Coronary artery disease; CFR: Coronary flow reserve; CHF: Chronic heart failure; CRT: Cardiac resynchronization therapy; DC: Dilated ischemic cardiomyopathy; DCM: Idiopathic dilated cardiomyopathy; DIP: Dipyridamole; DOB: Dobutamine; ΔESPVR (or simpler PVR pressure-volume relation): The difference between peak stress and rest ESPVR; EF: Ejection fraction; ESP: End-systolic pressure; ESPVR: End-systolic pressure-volume-ratio; EX: Exercise; FFR: Force-frequency relation; ICD: Implantable cardioverter defibrillator; LV: Left ventricle; LVESP: Left ventricular end-systolic pressure; MR: Mitral regurgitation; PM: Pace-maker; PVR: Pressure-volume relation; TX: Heart transplant; PCI: Percutaneous coronary intervention; 2-DE: Two-dimensional echo.

## Competing interests

The authors declare that they have no competing interests.

## Authors’ contributions

TB conceived this review, performed the data analysis, and drafted the manuscript; MZ and TL are chief and coworker of the Scientific Visualization Unit Group; QC, LC, EA, LP, were responsible for data collection and revised the manuscript; SS performed the statistical analysis; EP contributed to the preparation of review design, data discussion, and critical revision of the manuscript. All authors read and approved the final manuscript.

## Authors’ information

TB, Scientific Coordinator of the CCM project n. 48 “Aged Donor Heart Rescue by Stress Echo – ADONHERS” Institute of Clinical Physiology, National Research Council, Pisa, Italy. MZ, Director, Scientific Visualization Unit, Institute of Clinical Physiology, National Research Council, Pisa, Italy. QC, Division of Cardiology, Fatebenefratelli Hospital, Benevento, Italy. EA, Division of Noninvasive Cardiology, Cardiothoracic Department, San Raffaele Hospital, IRCCS, Milano, Italy. SS, PhD, Institute of Clinical Physiology, National Research Council, Pisa, Italy. TL, Scientific Visualization Unit, Institute of Clinical Physiology, National Research Council, Pisa, Italy. LP, Institute of Clinical Physiology, National Research Council, Pisa, Italy. EP, Acting Director Institute of Clinical Physiology, National Research Council, Pisa, Italy.

## Supplementary Material

Additional file 1 **Dilated Cardiomyopathy: A molecular view.** Dilated Cardiomyopathy is a common heart disease that causes heart myofibers to pulse in a weakened way, which becomes particularly dangerous under stress, when heartbeat is accelerated. (The full quality video can be accessed at this link: https://vimeo.com/79085937). Click here for file

Additional file 2 **Dilated Cardiomyopathy: The sarcomere At each cycle of heartbeat, the heart cell contracts (shortens) at systole and relaxes at diastole.** Under stress, both the speed and the intensity of the movement are increased. In the DCM heart, both the structure and the activity are impaired: the mitochondrion is enlarged, due to the excess ATP request, and the fibers are not as compact and aligned as in the healthy fiber. The relaxation phase is never complete, resulting in the typical ECG track. Under stress the situation is worsened: the heart fibers, unable to completely de-contract, try to compensate by even stronger contraction, in a forward loop that worsens with time, and can lead to heart failure. (The full quality video can be accessed at this link: https://vimeo.com/79085938). Click here for file

Additional file 3 **Dilated Cardiomyopathy: Ca**^**2+ **^**induced Ca**^**2+**^** release (zoom to synaptic junction).** The signal to initiate contraction is a brief depolarization of the membrane, which triggers a very rapid release of calcium from the sarcolemma. This is followed by more sustained release of Ca^**2+**^ from the ryanodine receptor on the T Tubules. In this way, the signal rapidly diffuses throughout the contractile fiber. When more blood is needed in the organism, the frequency of signal is increased, and more Ca^**2+**^ is released. However, all Ca^**2+**^ is removed from the fiber before the onset of next beat. The DCM heart is impaired in the re-uptake of Ca^**2+**^, resulting in a small amount of Calcium constantly present in the fiber. When more CA^**2+**^ is released because of higher demand, the problem of re-uptake worsens. (The full quality video can be accessed at this link: https://vimeo.com/79085979). Click here for file

Additional file 4 **Dilated Cardiomyopathy: Actin-myosin contraction.** Contraction is the result a sliding of actin and myosin fibers, due to the activity of the myosin cycle: this cycle is active in the presence of Calcium, which mediates transmission of signal to both the actin and myosin fibers, via other accessory proteins. The activity requires energy, in the form of ATP (not shown). When Calcium has been removed, the myosin heads detach from the actin fibers and the muscle can relax. In the DCM heart calcium re-uptake is incomplete: this leads to a constant contraction which, on one hand requires more energy (hence the enlarged mitochondria) and on the other impedes full extension of the heart fibers during relaxation, thus limiting the amount of incoming blood. The problem is even worse under stress conditions. Note that due to the excess work and the lack of total relaxation, the actin and myosin fibers of the dilated heart are not regularly aligned and the overall structure of the myofiber is slightly disorganized. (The full quality video can be accessed at this link: https://vimeo.com/79085977). Click here for file

Additional file 5 **Dilated Cardiomyopathy: Calcium re-uptake.** Clearing of intracellular Calcium is carried out by the Sarcoplasmic Reticulum Calcium Pump (known as SERCA), a very efficient protein capable of concentrating Calcium at a rate of two ions for one ATP. One of the molecular defects of the dilated heart is the reduced efficiency of this pump: this is physiologically counterbalanced by a higher expression (and more intense activation) of the Na^**+**^/Ca^**2+**^ exchanger of the plasma membrane. The problem is especially felt under stress: in this condition, with faster cycles, even more Calcium is released, aggravating the burden of re-uptake on the defective SERCA, not well-complemented by the activity of the plasma membrane exchanger. Since this pump is also inefficient, the final result is that blood circulation is not sustained to the required level. (The full quality video can be accessed at this link: https://vimeo.com/79085978). Click here for file

Additional file 6 **Force-frequency curve with stress echo in a normal subject.** Upper panel: On the left, systolic blood pressure by cuff sphygmomanometer (SP, first row); left ventricular end-systolic volumes calculated with biplane Simpson method (ESV, second row); heart rate increase during stress (bpm, third row); in the lowest row, the force-frequency curve built off-line with the values recorded at baseline (second column), and at different steps (third, fourth, fifth column) up to peak stress (sixth column). An increased heart rate is accompanied by an increased systolic pressure with smaller end-systolic volumes (normal up sloping force-frequency relation). Click here for file

Additional file 7 **Force-frequency curve with stress echo in a subject with dilated cardiomyopathy and depressed baseline left ventricular function (EF% = 30%).** On the left: systolic blood pressure by cuff sphygmomanometer (SP, first row); left ventricular end-systolic volumes calculated with biplane Simpson method (ESV, second row); heart rate increase during stress (bpm, third row); in the lowest row, the force-frequency relation built off-line with the values recorded at baseline (second column), and at different steps (third, fourth, fifth column) up to peak stress (sixth column). An increased heart rate at peak exercise is accompanied by no changes in end-systolic volumes (abnormal flat force-frequency relation). Click here for file
