# Supplementary material for: Myocardial contractility in the stress echo lab: from pathophysiological toy to clinical tool
Source: Cardiovasc Ultrasound. 2013 Nov 18;11:41. doi: 10.1186/1476-7120-11-41 (PMC3875530; doi:10.1186/1476-7120-11-41)
Supplement: Additional file 6 — Force-frequency curve with stress echo in a normal subject. Upper panel: On the left, systolic blood pressure by cuff sphygmomanometer (SP, first row); left ventricular end-systolic volumes calculated with biplane Simpson method (ESV, second row); heart rate increase during stress (bpm, third row); in the lowest row, the force-frequency curve built off-line with the values recorded at baseline (second column), and at different steps (third, fourth, fifth column) up to peak stress (sixth column). An increased heart rate is accompanied by an increased systolic pressure with smaller end-systolic volumes (normal up sloping force-frequency relation). [file 1476-7120-11-41-S6.pptx]

## Slide 1
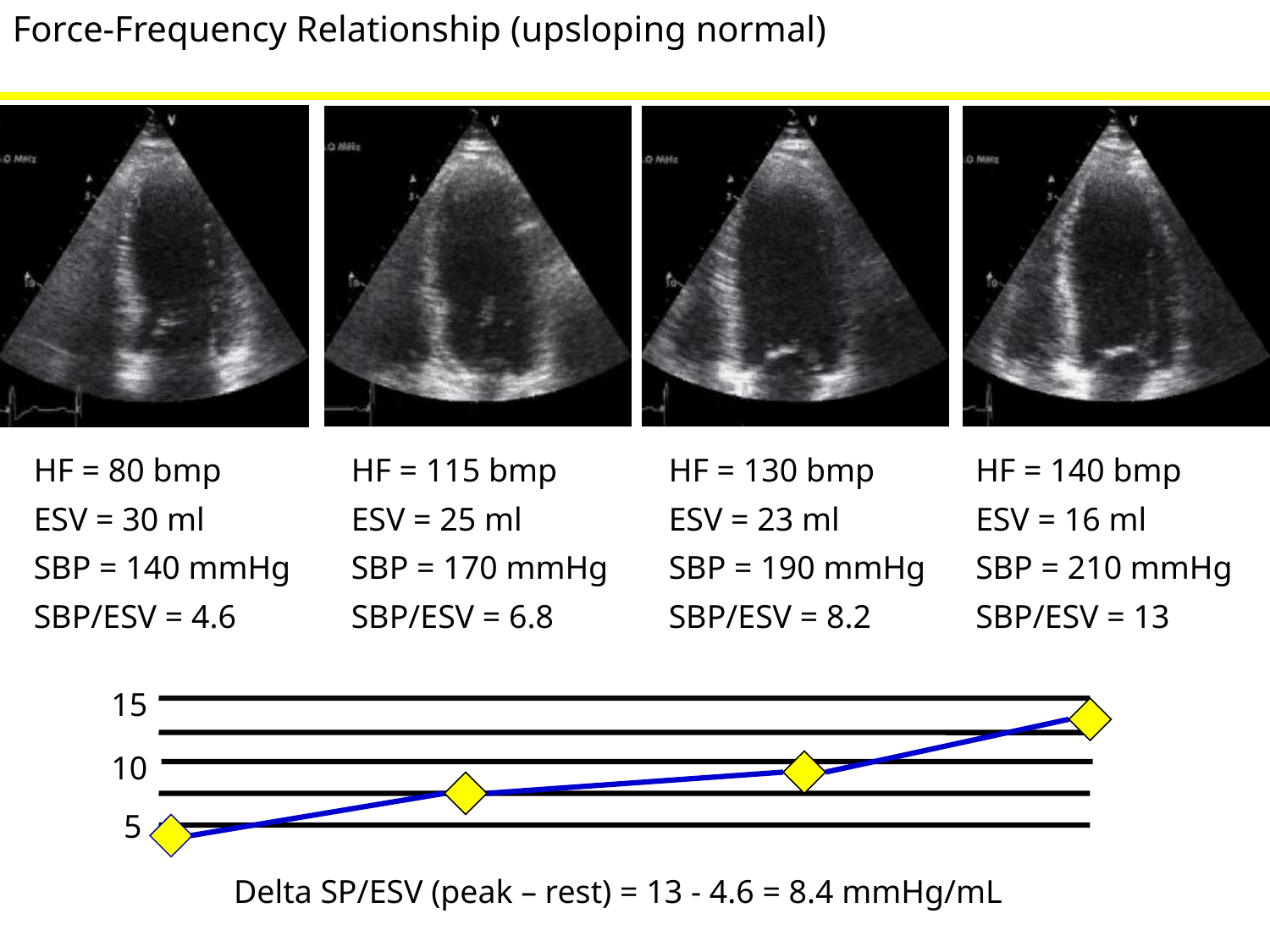

Force-Frequency Relationship (upsloping normal)
HF = 80 bmp
ESV = 30 ml
SBP = 140 mmHg
SBP/ESV = 4.6
HF = 115 bmp
ESV = 25 ml
SBP = 170 mmHg
SBP/ESV = 6.8
HF = 130 bmp
ESV = 23 ml
SBP = 190 mmHg
SBP/ESV = 8.2
HF = 140 bmp
ESV = 16 ml
SBP = 210 mmHg
SBP/ESV = 13
15
10
5
Delta SP/ESV (peak – rest) = 13 - 4.6 = 8.4 mmHg/mL
