# Supplementary material for: Myocardial contractility in the stress echo lab: from pathophysiological toy to clinical tool
Source: Cardiovasc Ultrasound. 2013 Nov 18;11:41. doi: 10.1186/1476-7120-11-41 (PMC3875530; doi:10.1186/1476-7120-11-41)
Supplement: Additional file 7 — Force-frequency curve with stress echo in a subject with dilated cardiomyopathy and depressed baseline left ventricular function (EF% = 30%). On the left: systolic blood pressure by cuff sphygmomanometer (SP, first row); left ventricular end-systolic volumes calculated with biplane Simpson method (ESV, second row); heart rate increase during stress (bpm, third row); in the lowest row, the force-frequency relation built off-line with the values recorded at baseline (second column), and at different steps (third, fourth, fifth column) up to peak stress (sixth column). An increased heart rate at peak exercise is accompanied by no changes in end-systolic volumes (abnormal flat force-frequency relation). [file 1476-7120-11-41-S7.pptx]

## Slide 1
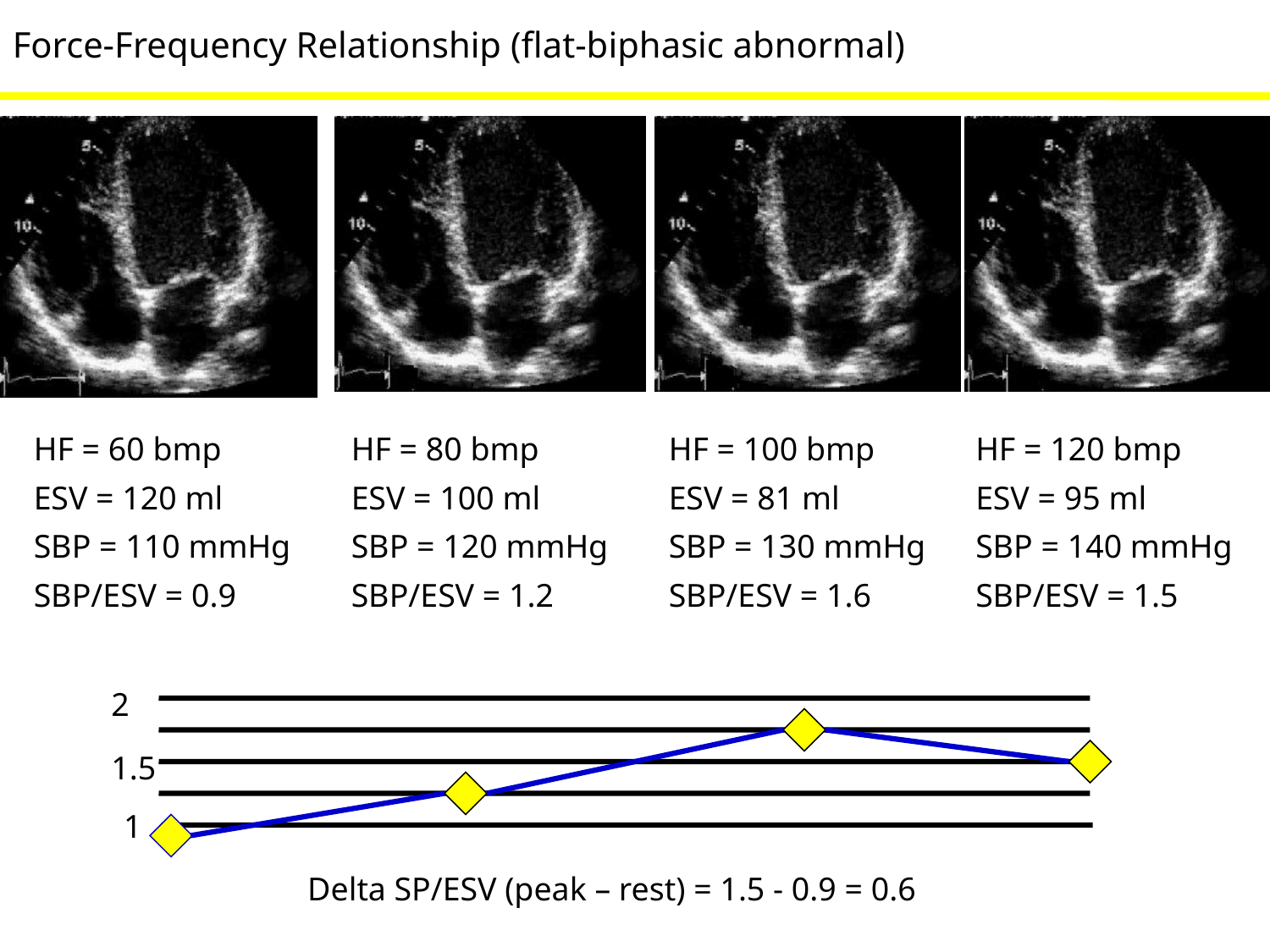

Force-Frequency Relationship (flat-biphasic abnormal)
HF = 60 bmp
ESV = 120 ml
SBP = 110 mmHg
SBP/ESV = 0.9
HF = 80 bmp
ESV = 100 ml
SBP = 120 mmHg
SBP/ESV = 1.2
HF = 100 bmp
ESV = 81 ml
SBP = 130 mmHg
SBP/ESV = 1.6
HF = 120 bmp
ESV = 95 ml
SBP = 140 mmHg
SBP/ESV = 1.5
2
1.5
1
Delta SP/ESV (peak – rest) = 1.5 - 0.9 = 0.6
